# How needs and preferences of employees influence participation in health promotion programs: a six-month follow-up study

**DOI:** 10.1186/1471-2458-14-1277

**Published:** 2014-12-15

**Authors:** Anne Rongen, Suzan J W Robroek, Wouter van Ginkel, Dennis Lindeboom, Martin Pet, Alex Burdorf

**Affiliations:** Department of Public Health, Erasmus MC, University Medical Center Rotterdam, PO Box 2040, 3000 CA Rotterdam, the Netherlands; Werkgeversforum Kroon op het Werk, 2132 JJ Hoofddorp, the Netherlands; WerkVanNu, 2726 VA Zoetermeer, the Netherlands; Lifeguard B.V, 3508 AE Utrecht, the Netherlands

**Keywords:** Workplace, Health promotion, Preferences, Health behavior, Participation

## Abstract

**Background:**

Low participation in health promotion programs (HPPs) might hamper their effectiveness. A potential reason for low participation is disagreement between needs and preferences of potential participants and the actual HPPs offered. This study aimed to investigate employees’ need and preferences for HPPs, whether these are matched by what their employers provide, and whether a higher agreement enhanced participation.

**Methods:**

Employees of two organizations participated in a six-month follow-up study (n = 738). At baseline, information was collected on employees’ needs and preferences for the topic of the HPP (i.e. physical activity, healthy nutrition, smoking cessation, stress management, general health), whether they favored a HPP via their employer or at their own discretion, and their preferred HPP regarding three components with each two alternatives: mode of delivery (individual vs. group), intensity (single vs. multiple meetings), and content (assignments vs. information). Participation in HPPs was assessed at six-month follow-up. In consultation with occupational health managers (n = 2), information was gathered on the HPPs the employers provided. The level of agreement between preferred and provided HPPs was calculated (range: 0–1) and its influence on participation was studied using logistic regression analyses.

**Results:**

Most employees reported needing a HPP addressing physical activity (55%) and most employees preferred HPPs organized via their employer. The mean level of agreement between the preferred and offered HPPs ranged from 0.71 for mode of delivery to 0.84 for intensity, and was 0.47 for all three HPP components within a topic combined. Employees with a higher agreement on mode of delivery (OR: 1.72, 95% CI: 0.87-3.39) and all HPP components combined (OR: 2.36, 95% CI: 0.68-8.17) seemed to be more likely to participate in HPPs, but due to low participation these associations were not statistically significant.

**Conclusion:**

HPPs aimed at physical activity were most needed by employees. The majority of employees favor HPPs organized via the employer above those at their own discretion, supporting the provision of HPPs at the workplace. This study provides some indications that a higher agreement between employees’ needs and preferences and HPPs made available by their employers will enhance participation.

**Electronic supplementary material:**

The online version of this article (doi:10.1186/1471-2458-14-1277) contains supplementary material, which is available to authorized users.

## Background

Workplace health promotion programs (WHPPs) are increasingly being provided to employees, especially in larger organizations [1]. Such programs have shown to improve employees’ lifestyle (e.g. physical activity, nutrition) [2–4]. Moreover, WHPPs may increase employees’ productivity at work and decrease their sickness absence [5–8]. However, effect sizes (ES = 0.24) of WHPPs are often modest [9], and although most employees are interested in WHPPs [10] few actually participate [11]. Since low participation limits the potential effectiveness of WHPPs, it is essential to study how participation can be enhanced [12].

The effectiveness of WHPPs as well as participation in WHPPs differ by demographic and intervention characteristics. Female employees are more inclined to participate and WHPPs among younger employees show greater effects [9, 11]. Higher participation is reached with WHPPs focusing on multiple behaviors and consisting of various components [11] and effectiveness of WHPPs is greater when it consists of multiple meetings [9]. Hence, participation in WHPPs and its effectiveness depend partly on the characteristics of the study population and the design of the WHPP. Furthermore, barriers related to the individual (e.g. no time, no motivation) as well as logistic reasons (e.g. location and time of the program) during implementation are often said to impede participation [13–17]. Although these barriers lower the likelihood of employees having a positive intention towards participation, they hardly influence their decision to actually participate [18]. So, more insight is needed into other factors that might explain participation.

Frameworks like the ‘intervention mapping’ protocol [19] and ‘precede-proceed’ model [20] emphasize the importance of a needs assessment for developing health promotion programs (HPPs) that are attractive and address the needs and preferences of the target population. Hence, a disagreement between the needs and preferences of the target population and the HPPs provided might lower participation. Studies on preferences for HPPs are often qualitative or limited to a HPP developed for a specific research purpose [21–24]. There is a lack of quantitative studies investigating HPP needs and preferences in general. Furthermore, it is unknown whether the degree to which individuals’ preferences are met will actually enhance participation. At the workplace, low participation might be due to a mismatch between the needs and preferences of the employees and the HPPs provided by their employers.

This study aimed to investigate employees’ need and preferences with regard to HPPs, whether these are matched by what their employers provide, and whether a higher level of agreement enhances participation.

## Methods

### Study population

The population in this six-month follow-up study consisted of employees of a plastics manufacturer (organization 1, n = 874) and a paint manufacturer (organization 2, n = 1281). Both organizations had in place a variety of HPPs that were accessible for all employees.

Between 2010 and 2012, all employees were invited by e-mail to fill in two online questionnaires: a baseline questionnaire and a follow-up questionnaire six months later. For this study, we included all employees who completed both the baseline and follow-up questionnaires.

Of the 2155 employees invited, 1128 (52%) completed the baseline questionnaire. Of this group, 761 (68%) also completed the follow-up questionnaire after six months and 748 employees (98%) provided informed consent. Four employees were excluded due to implausible or missing data on height, weight, or physical activity, and six employees because of incomplete information on HPP preferences. The final study sample comprised 738 employees (organization 1, n = 276; organization 2, n = 449).

Informed consent was requested at the start of the questionnaire. The Medical Ethical Committee of Erasmus MC (Rotterdam, the Netherlands) declared that the Medical Research Involving Human Subjects Act did not apply to the current study and the committee had no objection to the execution of this study.

### Data collection

#### Participation in a HPP

At six-month follow-up, employees were asked whether or not they had actually participated in a HPP during the six-month follow-up period. A HPP was defined in the questionnaire as follows: “A program that is aimed at improving your health behavior. For example, smoking cessation program, fitness participation, participating in a meeting on healthy nutrition”. HPPs could either be organized by the employer or take place outside the workplace and be organized by the employee themselves (referred to as ‘own discretion’).

#### HPP preferences

At baseline, all employees were asked about their needs and preferences with regard to HPPs. The first question asked about the topic the HPP needed to address, distinguishing physical activity, healthy nutrition, smoking cessation, stress management, and general health (“When you would participate in a health promotion program, what should it be aimed at?”). Every employee was asked to choose at least one topic but multiple topics were permitted. A summation was calculated for the number of HPP topics the employee indicated. Per topic, employees were asked whether they favored a HPP that was organized via their employer or at their own discretion. Additionally, per topic, employees were asked about what HPP they preferred with regard to three components with each two alternatives: mode of delivery (individual versus group program), intensity (single meeting versus multiple meetings), and content (provide information versus assignments).

#### HPPs offered by employers

In consultation with the occupational health managers (n = 2), we collected information on the HPPs the organizations provided. We specifically asked about the HPPs they provided that focused on physical activity, healthy nutrition, smoking cessation, stress management, or improving general health. Examples of HPPs the organizations offered are a fitness center on site or appointments with a dietician (Additional file 1: Table S1). We categorized all provided HPPs according to the three components of the design of the HPP (i.e. mode of delivery, intensity, and content) (Additional file 1: Table S2).

#### Level of agreement

By comparing employees’ preferences for a HPP within a specific topic with the HPPs employers provided on that topic we assessed whether there was a match with regard to three components of the HPP (i.e. mode of delivery, intensity, content) per topic. Furthermore, the percentage of ‘overall agreement’ was calculated per HPP topic. This indicates the percentage of employees for whom their preferred HPP matched on all three components with what their employer provided.

Three different levels of agreement were calculated. The first level of agreement was assessed for each of the three components (i.e. mode of delivery, intensity, content) across all topics. This first level of agreement indicated the number of agreements between employees’ preferences for the specific component and that of the HPPs provided expressed by the number of preferences, taking into account the number of topics an employee had indicated. The second level of agreement was calculated across all HPP topics and all components. It is the aggregated measure of the first levels of agreement and is referred to as all component agreement. The third level of agreement was calculated across all components per HPP topic. It assessed the agreement between employees’ preference for a particular combination of components for a specific topic with the characteristics of the HPPs provided. This level of agreement is the strictest measure and is referred to as complete program agreement. All levels of agreement have a score ranging from 0 (no agreement at all) to 1 (perfect agreement).

#### Self-perceived health and health behavior

Self-perceived health was measured using the first question on the Short Form-12 (SF-12) questionnaire (“Overall, how would you rate your health during the past 4 weeks?”). The five possible answers were dichotomized into ‘poor or fair’ and ‘good, very good, or excellent’ [25].

Body mass index (BMI: weight/height^2^) was calculated based on self-reported height in meters and weight in kilograms and categorized into normal weight (BMI < 25 kg/m^2^), overweight (25 ≤ BMI < 30 kg/m^2^), and obesity (BMI ≥ 30 kg/m^2^).

Physical activity was measured using a slightly adapted version of the short form International Physical Activity Questionnaire (IPAQ) [26], which measures physical activity of moderate intensity. Questions on walking were excluded since walking at a low pace is considered to be of low intensity. The average amount of leisure time spent on moderate intensity physical activity was calculated as follows: employees were first asked how many days per week they engaged in moderate intensity physical activity; they were then asked how many minutes on average was spent on moderate intensity physical activity, per occasion. Dichotomization was based on recommendations for moderate intensity physical activity that requires such levels of activity for at least 30 minutes per day [27]. Employees who were physically active at a moderate intensity level for at least 210 minutes a week (7 times 30 minutes) were considered to have met this recommendation.

Fruit and vegetable intake was measured using a slightly adapted version of the Dutch Food Frequency Questionnaire [28]. The six-item questionnaire asked about the monthly intake of different fruits (four items, e.g. apple, fruit juice) and vegetables (two items: raw and cooked vegetables). Dichotomization was based on the Dutch guidelines for healthy nutrition that states that one should to consume 200 grams of fruit and 200 grams vegetables daily. Employees who ate at least 400 grams of fruit and vegetables per day were considered to meet the guidelines.

Smoking was assessed using a single-item question: “Do you smoke?”. Answer possibilities were: ‘yes’, ‘now and then’, and ‘no’. Employees answering the question with ‘yes’ or ‘now and then’ were defined as being a ‘current smoker’.

#### Individual characteristics

The following individual characteristics were assessed: age, gender, and educational level. Age was categorized into three groups: 18–39, 40–49, and 50–65. Educational level was determined by asking the employees about their highest level of education, which was then categorized as follows: low (primary school, lower and intermediate-level secondary schooling, or lower vocational training); intermediate (higher-level secondary schooling or intermediate vocational training); and high (higher vocational training or university).

### Data analysis

Descriptive statistics were used to report on the following: the characteristics of the study population, the topic the HPP needed to address according to the employees, whether employees favored the HPP to be organized by their employer or at their own discretion, the preferred HPP per topic with regard to the three components (i.e. mode of delivery, intensity, content), and the levels of agreement between the preferred HPP and those provided by the employer.

First, logistic regression analysis were used to assess whether selective loss to follow-up had occurred. Second, logistic regression analyses were used to study associations between individual characteristics (age, gender, educational level) and the five needed topics of the HPP (i.e. physical activity, healthy nutrition, smoking cessation, stress management, general health). Third, logistic regression analyses were used to study how the health behaviors of the employees were associated with the corresponding topic of the HPP. Last, logistic regression analyses, adjusted for individual characteristics, were used to study associations between the three different levels of agreement and participation in a HPP. In these analyses, the level of agreement was entered as a continuous variable.

The odds ratio (OR) was estimated as measure of association with a corresponding 95% confidence interval (95% CI). All analyses were carried out using the IBM SPSS Statistics version 20 for Windows (SPSS Inc., Chicago, IL, USA).

## Results

### Description of the study population

The study population consisted of 738 employees with a mean age of 44.9 years (SD: 9.3) and mean BMI of 25.6 kg/m^2^ (SD: 3.6). Further details are presented in Table 1.

**Table 1:** **The characteristics of the study population (n = 738)**

	n	%
Individual characteristics		
Age		
18-39	217	29.4
40-49	268	36.3
50-65	253	34.3
Male	544	73.7
Educational level		
Low	142	19.2
Intermediate	199	27.0
High	397	53.8
Health behaviors		
Body Mass Index (BMI)		
Normal weight (BMI < 25 kg/m^2^)	358	48.5
Overweight (25 ≤ BMI < 30 kg/m^2^)	297	40.2
Obese (BMI 30 kg/m^2^ and higher)	83	11.2
Insufficient moderate intensity physical activity (less than 30 min a day)	371	50.3
Insufficient fruit and vegetable intake (less than 400 grams a day)	489	66.3
Current smoker	140	19.0
Self-perceived health		
Less than good self-perceived health	33	4.5
Participation in a health promotion program		
Participation during six-month follow-up period	83	11.2

The percentage of employees aged 50 years or older was higher in the group who completed both questionnaires than in the group who completed only the baseline questionnaire (34% versus 26%), other individual characteristics were similarly distributed. Employees lost to follow-up did not differ from those completing both questionnaires with regard to self-perceived health and health behavior. Fewer employees who completed both questionnaires had a preference for a smoking cessation program (7% versus 11%).

### Health promotion program preferences

More than half of the employees (55%) reported to need a HPP that addresses physical activity, followed by general health (45%), stress management (39%), healthy nutrition (33%), and smoking cessation (7%). About half of the employees (47%) indicated needing only one topic to be addressed by a HPP, 32% of the employees indicated two topics, and 21% three or more topics.

In general, most employees favored HPPs organized by their employer rather than those at their own discretion (59%). Across all topics, employees preferred HPPs that had an individual focus (67%) and HPPs that consisted of multiple meetings (62%). For HPPs that address physical activity or stress management, employees favored that the HPP gave assignments. For HPPs addressing the other topics (i.e. healthy nutrition, smoking cessation, and general health) employees favored HPPs that provide information (Table 2).

**Table 2:** **Preferences for health promotion programs among 738 employees**

	Setting	Mode of delivery	Intensity	Content
	**Offered by employer rather than at own discretion**	**Individual rather than group**	**Multiple meetings rather than once**	**Assignments rather than information**
Topic				
Physical activity (n = 406)	58%	64%	69%	71%
General health (n = 334)	59%	64%	47%	32%
Stress management (n = 290)	58%	70%	69%	56%
Healthy nutrition (n = 240)	62%	74%	60%	43%
Smoking cessation (n = 51)	63%	65%	65%	37%

The need for a specific HPP topic differed by individual characteristics. HPPs addressing physical activity (18–39: OR: 1.58, 95% CI: 1.09-2.28), healthy nutrition (18–39: OR: 1.99, 95% CI: 1.34-2.96; 40–49: OR: 1.73, 95% CI: 1.18-2.53), and stress management (18–39: OR: 1.82, 95% CI: 1.25-2.64) were more often needed by younger employees, while HPPs focusing on general health were particularly requested by older employees (50–64: OR: 1.76, 95% CI: 1.21-2.54). Needs for HPPs on physical activity (intermediate: OR: 1.54, 95% CI: 1.00-2.38; high: OR: 1.52, 95% CI: 1.04-2.24) and stress management (intermediate: OR: 2.47, 95% CI: 1.53-3.97; high: OR: 2.35, 95% CI: 1.53-3.63) were more often expressed by higher educated compared to lower educated employees, while smoking cessation HPPs were mainly requested by lower (OR: 2.64, 95% CI: 1.28-5.44) and intermediate (OR: 2.36, 95% CI: 1.20-4.65) educated employees. Only for stress management HPPs there was a gender difference, with more female than male employees needing HPPs focusing on this topic (OR: 2.36, 95% CI: 1.69-3.29).

Employees being insufficiently physical active on a moderate intensity were more likely to indicate a need for a HPP addressing physical activity (OR: 1.45, 95% CI: 1.08-1.94) and employees who currently smoked were more likely to express needing a smoking cessation HPP (OR: 58.04, 95% CI: 22.49-149.81). For all other HPP topics no statistically significant associations were found between employees’ health behavior and the corresponding HPP (data not shown).

### Agreement between preferred and offered HPPs

Table 3 shows the degree of agreement for the 15 comparisons between the preferences of employees with regard to HPPs and what their employer provided. For five comparisons, all preferences were matched by the HPPs the employer provided. For the other comparisons, the degree of agreement ranged between 31% and 86% with a mean degree of agreement of 70%. The preferred HPP matched on all three components with what the organizations provided for 24% of the employees who needed a smoking cessation HPP to 69% for employees needing a physical activity HPP (Table 3).

**Table 3:** **Agreement (%) between preferred and offered health promotion programs (HPPs) among 738 employees, stratified by topic**

Components	Mode of delivery	Intensity	Content	Complete program
Topic				
Physical activity (n = 406)	100%	69%	100%	69%
General health (n = 334)	64%	100%	67%	45%
Stress management (n = 290)	31%	91%	86%	26%
Healthy nutrition (n = 240)	83%	100%	57%	46%
Smoking cessation (n = 51)	35%	27%	43%	24%

The mean level of agreement on the three components of the HPPs varied from 0.71 (SD: 0.37) for mode of delivery to 0.84 (SD: 0.31) for intensity. The mean level of agreement on all components was 0.78 (SD: 0.20) and that of the complete program was 0.47 (SD: 0.41) (Table 4).

**Table 4:** **The influence of levels of agreement on participation in HPP among 738 employees**

	Level of agreement	Participation in HPP (n = 83)
	**Mean (SD)**	**OR (95% CI)**
Level of agreement on		
Mode of delivery (0–1)	0.71 (0.37)	1.72 (0.87-3.39)
Intensity (0–1)	0.84 (0.31)	1.19 (0.55-2.58)
Content (0–1)	0.80 (0.33)	1.12 (0.55-2.28)
All components (0–1)	0.78 (0.20)	2.36 (0.68-8.17)
Complete program (0–1)	0.47 (0.41)	0.99 (0.57-1.74)

Note: the analyses are adjusted for age, gender, and educational level, the level of agreement is a continuous variable, therefore the OR indicates the increase in odds by an increase in agreement.

HPP: health promotion program.

### Influence of the levels of agreement on participation

Employees who indicated a need for at least two topics to be addressed by a HPP were not more likely to participate in a HPP as compared to those employees who reported a need for one topic (OR: 1.30, 95% CI: 0.82-2.07). The influence of the level of agreement on the separate components of the HPP (the first level of agreement) on actual participation ranged from OR = 1.12 (95% CI: 0.55-2.28) for content to OR = 1.72 (95% CI: 0.87-3.39) for mode of delivery. Employees with a higher agreement on all components combined seemed to be more likely to participate (OR: 2.36, 95% CI: 0.68-8.17). However, this association was not statistically significant. Agreement on all components within a topic between the preferred HPP and the HPP provided by the employers, the third level of agreement, did not enhance participation (OR: 0.99, 95% CI: 0.57-1.74) (Table 4). Age, gender, and educational level were not statistically significantly associated with participation (ORs close to unity) (data not shown).

## Discussion

Most employees needed a HPP aimed at improving physical activity. HPPs organized via the employer were favored rather than those at employees’ own discretion. The preferred HPP for addressing physical activity had the highest agreement with the HPPs the employers provided, followed by HPPs on healthy nutrition, general health, stress management, and smoking cessation. The mean level of agreement for the HPP components (i.e. mode of delivery, intensity, and content) ranged from 0.71 (mode of delivery) to 0.84 (intensity) with an agreement of 0.47 for the complete HPP. The results provided some indications that employee’s with a higher agreement between their preferences and what their employer provided were more likely to participate in HPPs.

Physical activity is the most needed topic for HPP according to the respondents. The popularity of physical activity HPPs was also observed by Persson and colleagues (2014) [29]. In their study, 46% of the employees expressed that they were willing to change their health behavior in relation to physical activity. Furthermore, in a recent systematic review on WHPPs aimed at a healthy lifestyle, the majority (61%) of the included WHPPs focused on improving physical activity [9]. Our finding that most employees favored a HPP organized by their employer corroborates earlier findings [10, 30] and supports the provision of HPPs at the workplace. It emphasizes the need to develop effective WHPPs attractive to employees. A concern in workplace health promotion is whether those employees are reached who would benefit most by participating in HPPs [31, 32]. Previous studies showed mixed results on this issue [33, 34]. We found that those employees who were insufficiently physically active were more likely to prefer a HPP aimed at increasing physical activity. However, for the other health behaviors no association was found between unfavorable health behaviors and an expressed need for a HPP addressing that health behavior.

The needed topic of the HPP differed between demographic groups. Younger employees more often stated needing a HPP focusing on physical activity, healthy nutrition, and stress management while older employees wanted HPPs that addressed general health. The latter might reflect the higher prevalence of common health problems and chronic diseases at older age. Due to the differences in the needs of employees, it is recommended to conduct needs assessments and tailor interventions to the characteristics of the target population. Studies incorporating these methods have shown to lead to more positive results with regard to program effectiveness and appreciation [35–37]. Moreover, performing subgroup analysis in future research may expand our knowledge on who participates in which types of WHPP.

Previous studies investigating preferences for health promotion were often qualitative or they studied preferences with regard to the development of a specific program [21–24]. Blackford et al. [13] is one of the few investigating preferred strategies for WHPPs in general and reported that most employees preferred a stretching program at their desk and personalized dietary recipes. However, the preferred HPPs were not implemented and therefore they could not study whether employees were indeed going to participate in the preferred programs. The our study, the needs and preferences of employees for HPPs were investigated using questionnaires. In addition, we studied how well the HPPs the organizations provided matched the preferences of the employees. For HPPs on physical activity there was a high agreement between preferred HPPs and those provided (69%). This was due to the great diversity of physical activity programs the employers provided. Smoking cessation programs had the lowest agreement, but since only one organization provided such programs this result is distorted. For the organization that did provide smoking cessation programs, the level of agreement was much higher (55%). The limited provision of HPPs on smoking cessation may reflect a shift towards attention for implementing smoking bans. Employers in the Netherlands are since 2004 obliged by law to provide smoke-free workplaces.

Some indications were found that employees with a higher level of agreement on the way a program was delivered were more likely to participate in a HPP. For the participating organizations, there was a relatively high agreement on this component for HPPs addressing physical activity, healthy nutrition, and general health. However, for stress management HPPs there seems to be a lack of individually based HPPs. The associations of agreement on the specific components (second level of agreement) and on the complete program (third level of agreement) with participation in HPPs revealed that employees were more likely to participate when their preferences for a HPP matched with the HPPs provided by their employers. However, a match on all three components of the HPP within a topic did not enhance participation. Hence, it seems that agreement on all HPP components is unnecessary for participation. Since agreement on the mode of delivery had the highest odds for participation, we assume that a match on this component is most important for participation.

Due to a limited number of employees actually participating in HPPs (11%) the associations between the levels of agreement and participation were not statistically significant and should therefore be interpreted with caution. However, the ORs of the specific components as well as of all components combined are in the same direction. It would be interesting to investigate in a larger cohort whether our results are corroborated. In future research, it might also be interesting to question the preferred delivery of the content (e.g. through the internet, face-to-face) and to study whether a better fit between preferred and provided HPPs will lead to greater effectiveness. Moreover, previous research showed that the physical and social environment, incentives, time constraints, management support, and the possible outcome achieved could influence participation [12, 38–43]. In future research it would be interesting to investigate whether these factors modify the observed influence of workers’ preferences on participation.

### Strengths and limitations

As far as we know this is the first study investigating the influence of the level of agreement between employees’ preferences for a HPP and the HPPs provided by employers on participation in HPPs. A strength of this study is the follow-up design. Therefore, we could assess actual participation instead of intention to participate, which is often used as a proxy for participation [44]. However, a positive intention does not always result in actual participation [18]. In addition, by using a follow-up design reversed causality is less likely whereby participation in a HPP will influence preferences for a specific program, which were questioned in the baseline questionnaire. However, the short follow-up period of the study may have resulted in the limited number of employees who actually participated in a HPP and, consequently, in a lack of power. Furthermore, concerning reporting and selection bias, no statistically significant differences were found on gender, educational level, health behaviors, and self-perceived health between employees who completed both questionnaires and those lost to follow-up. Last, since all participants were employed in the manufacturing industry, the generalizability of the findings to other sectors of industry may be questioned. Nonetheless, employees with a variety of jobs were enrolled into the study. Future research is advised to include repeated measurements over a longer follow-up period and include a variety of organizations to increase statistical power and generalizability of the results. Furthermore, with a larger study population, it may also be possible to perform additional analyses such as stratification by new participants and employees who already participated in a HPP.

## Conclusion

HPPs aimed at improving physical activity were most needed by employees. The majority of employees favored HPPs that were organized by their employer above those at their own discretion. This supports the implementation of HPPs at the workplace. Some indications were found that agreement between preferences of employees regarding HPP components and the HPPs employer provide will increase participation. More research, in a larger cohort and a diversity of companies, is needed to assess whether our findings are corroborated in other populations.

## Electronic supplementary material

Additional file 1: Table S1: Health promotion programs the employers provided. **Table S2.** Classification of the health promotion programs the employers provided according to the three components of the HPPs. (DOCX 18 KB)
