# Supplementary material for: How needs and preferences of employees influence participation in health promotion programs: a six-month follow-up study
Source: BMC Public Health. 2014 Dec 15;14:1277. doi: 10.1186/1471-2458-14-1277 (PMC4301819; doi:10.1186/1471-2458-14-1277)
Supplement: Supplementary file 1 — Additional file 1: Table S1: Health promotion programs the employers provided. Table S2. Classification of the health promotion programs the employers provided according to the three components of the HPPs. (DOCX 18 KB) [file 12889_2014_7420_MOESM1_ESM.docx]

**Additional file 1**

**Table S1:** Health promotion programs the employers provided

|  | Organization 1 | Organization 2 |
| --- | --- | --- |
| Physical activity | A fitness facility on site offering individual a group programs. And a physiotherapist offering preventive programs. | A fitness school subscription with individual programs and group activities.  And a physiotherapist offering preventive programs. |
| Nutrition | A dietician on site. And a group program ‘do I eat healthy’ | Dietician on site |
| Smoking cessation | Individual smoking cessation coaching program | None |
| Stress management | Mediation and mindfulness in a group setting | A social worker giving information in a group setting |
| General health | A occupational physician on site | A occupational physician on site |

**Table S2:** Classification of the health promotion programs the employers provided according to the three components of the HPPs

| **Lifestyle targeted** | **Components** | **Organization 1** | **Organization 2** |
| --- | --- | --- | --- |
| Physical activity | Mode of delivery | 3 | 3 |
|  | Intensity | 2 | 2 |
|  | Content | 3 | 3 |
| Healthy nutrition | Mode of delivery | 3 | 1 |
|  | Intensity | 3 | 3 |
|  | Content | 1 | 1 |
| Smoking cessation | Mode of delivery | 1 | 0 |
|  | Intensity | 2 | 0 |
|  | Content | 3 | 0 |
| Stress management | Mode of delivery | 2 | 2 |
|  | Intensity | 2 | 3 |
|  | Content | 2 | 3 |
| General health | Mode of delivery | 1 | 1 |
|  | Intensity | 3 | 3 |
|  | Content | 1 | 1 |

- Mode of delivery: 0 = none, 1 = alone, 2= group, 3 = both options
- Intensity: 0 = none, 1 = once, 2= more meetings, 3 = both options
- Content: 0 = none, 1 = information, 2 = assignments, 3 = both options
